# Assessment of mid-term success in various types of zirconia implants: a retrospective clinical analysis

**DOI:** 10.1007/s00784-026-06751-9

**Published:** 2026-01-22

**Authors:** Belir Atalay, Yusuf Emes, Halim Işsever, Alanur Şahabettinoğlu, Buket Aybar, Tan Fırat Eyüboğlu, Mutlu Özcan

**Affiliations:** 1 Department of Oral and Maxillofacial Surgery, Faculty of Dentistry, Istanbul University, Istanbul, Türkiye; 2 Department of Public Health, Faculty of Medicine, Istanbul University, Istanbul, Türkiye; 3 Institute for Graduate Studies in Health Sciences, Oral and Maxillofacial Surgery PhD program, Istanbul University, Istanbul, Türkiye; 4 Faculty of Dentistry, Department of Endodontics, Istanbul Medipol University, Atatürk Bulvari, No: 27, Cibali Mahallesi, Istanbul, 34083 Türkiye; 5 Clinic of Masticatory Disorders and Dental Biomaterials, Center for Dental Medicine, University of Zurich, Zurich, Switzerland

**Keywords:** Clinical trial, Dental materials, Guided bone regeneration, Implant survival, One-piece implants, Two-piece implants, Zirconia implants

## Abstract

**Objectives:**

To evaluate the clinical outcomes of zirconia dental implants over five years, considering the growing demand for metal-free alternatives to titanium due to aesthetic concerns and potential hypersensitivity reactions to metals.

**Materials and methods:**

This retrospective study analyzed 80 zirconia implants from an initial cohort of 95, with 15 excluded due to loss to follow-up or not meeting inclusion criteria. Both one-piece and two-piece designs were included, and some cases involved Guided Bone Regeneration (GBR). The primary outcome was implant survival.

**Results:**

The overall survival rate was 83.8% (67/80) after a mean follow-up of 29.0 ± 16.6 months (maximum 62 months). Kaplan–Meier analysis estimated mean survival at 52.6 ± 2.38 months, with the median not reached due to censoring. Survival was significantly lower with immediate placement (*p* = 0.016). GBR was linked to higher survival, with all GBR implants surviving and failures only in non-GBR cases (*p* < 0.001). No significant differences appeared in implant design, loading protocol, diameter, jaw location, prosthesis, surface, zirconia brand, or patient sex.

**Conclusions:**

Zirconia implants showed 83.8% survival at five years, supporting their clinical applicability as metal-free implant systems*.* Immediate placement had significantly lower survival, favoring late placement. No significant differences were found between one-piece and two-piece implants, or between implants placed with and without GBR. Larger prospective studies with standardized clinical and radiographic assessments are needed to confirm these findings and refine zirconia implant protocols.

**Clinical Relevance:**

Zirconia implants show good mid-term survival and can be an alternative to titanium. However, immediate placement should be approached cautiously due to potential impacts on success.

## Introduction

Dental implants are widely recognized as a crucial treatment option for restoring missing teeth. Currently, pure titanium or titanium alloy is the gold standard in implant dentistry. Titanium has proven to be biocompatible and boasts high rates of survival and success [1–3]. However, despite titanium implants being biocompatible, esthetics remain a primary concern in the anterior region. Discoloration of the peri-implant soft tissues may occur due to a thin gingival phenotype. Another drawback of titanium is its potential to elicit allergic reactions [4]. Recently, the increased demand for metal-free restorations has sparked interest in alternative materials. Zirconium, a gray-white metal obtained through mining, is different from zirconia, a ceramic made of zirconium oxide [5].

The clinical application of zirconia has increased in prevalence over the past decade. Zirconia ceramics have a tooth-like color and exhibit favorable mechanical properties [6]. Nevertheless, zirconia is not without drawbacks. Concerns arise regarding its susceptibility to aging and degradation at lower temperatures. Material aging may occur due to induced stresses and microcracking [7].

Zirconia dental implants are primarily available in two configurations: one-piece or two-piece implants. Notably, one-piece zirconia implants are recognized as the preferred choice in clinical practice [8, 9]. Zirconia implants lack the extensive background research that titanium implants have. One-piece zirconia implants have been mostly documented, which show higher fracture resistance compared to two-piece zirconia implants [10]. When comparing titanium and zirconia implants, zirconia exhibits decreased bacterial plaque accumulation compared to titanium alloys, indicating reduced bacterial adhesion, which is crucial in the onset of peri-implant mucositis and peri-implantitis [11, 12]. However, titanium implants have been shown to provide higher long-term success rates, with studies demonstrating survival rates exceeding 95% after 10 years [1, 3].

This retrospective study sought to evaluate the mid-term success and survival rates of one-piece and two-piece zirconia dental implants over a five-year period. The analysis examined the influence of various clinical parameters, including guided bone regeneration, immediate placement, immediate loading, and implant diameter, on these survival rates. The null hypothesis of this study posited that there would be no statistically significant differences in survival rates between one-piece and two-piece zirconia implants. Additionally, it maintained that there would be no significant differences among implants placed with or without guided bone regeneration, immediate placement, immediate loading, or variations in implant diameter.

## Materials and methods

For this study, the findings from five years of follow-up on 95 zirconia implants placed between 2018 and 2023 were screened. After accounting for 15 implants lost to follow-up or excluded according to the study criteria, 80 implants remained available for analysis. Informed consent was obtained from all patients participating in the study. The study (Protocol No: 2023/26 Rev-2) received approval from the Istanbul University Faculty of Dentistry Clinical Research Ethics Committee.

The inclusion criteria for patients in this study were as follows: individuals aged 18 years or older, classified as ASA-1 or ASA-2, demonstrating good overall health, and capable of undergoing surgical treatment. Additionally, participants had to be free from systemic conditions that could impair bone healing around implants.

Exclusion criteria included heavy smoking (more than 20 cigarettes per day), pregnancy, and bruxism. Regarding implant-specific exclusions, implants with insertion torque values below 35 Ncm or ISQ scores below 55 were not considered eligible. Furthermore, implant sites exhibiting bone dehiscences, peri-implant bone defects exceeding 2 mm, less than 2 mm of bone beyond the root apex, or signs of acute infection were also excluded.

The primary outcome measure for implant success was defined as implant survival at the 5-year follow-up, which included the absence of implant mobility, suppuration, continuous peri-implant radiolucency, or need for implant removal.

### Surgical protocol

From the first day of operation until three days after implant placement, patients took Amoxicillin/clavulanic acid 875 mg/125 mg (Augmentin BID; GlaxoSmithKline, Istanbul, Türkiye), two tablets twice daily. Pain control was provided with a nonsteroidal anti-inflammatory drug, Diclofenac potassium 50 mg (Cataflam; Novartis, Istanbul, Türkiye). Following the surgical intervention, the patients were instructed to rinse with a 0.2% Chlorhexidine gluconate 0.2% mouthrinse (Klorhex; Drogsan, Ankara, Türkiye).

### Statistical analysis

Data was analyzed using IBM SPSS Statistics for Windows, Version 20 (Released 2011; IBM Corp., Armonk, New York, United States). A 95% confidence interval was established, and a p-value of less than 0.05 indicated statistically significant results in a two-tailed context. Descriptive statistics, such as mean, standard deviation, median, and minimum and maximum values, are employed for continuous data. For discrete data, both counts and percentages are calculated. The Kolmogorov-Smirnov and Shapiro-Wilk tests were utilized to assess the normality of the data distribution. The independent t-test was applied to parametric data for group comparisons, while the Mann-Whitney U and Chi-Square tests were utilized for non-parametric data analysis. The Chi-Square Test of Independence was conducted to ascertain potential associations between categorical variables.

## Results

A total of 95 implants were placed, of which 15 were excluded or lost to follow-up, leaving 80 implants for analysis. The demographic data is provided in Table 1.

**Table 1:** Survival outcomes of zirconia implants according to clinical and implant-related variables over a 5-year follow-up (*n* = 80 valid implants)

	Category	% of Implants	Number Placed	Number Failed	Survival Rate	Log-rank *p*-value
Placement timing	Immediate	53.8%	43	11	74.42%	**0.016**
	Late	46.2%	37	2	94.59%	
Loading protocol	Immediate	41.3%	33	6	81.82%	0.674
	Late	58.7%	47	7	94.59%	
GBR (overall)	Performed	13.75%	11	0	100%	**< 0.001**
	Not performed	86.25%	69	13	81.15%	
	One-piece	27.5%	22	4	81.82%	0.786
	Two-piece	72.5%	58	9	84.48%	
Implant width	Narrow	8.8%	7	0	100%	0.234
	Standard	91.2%	73	13	82.19%	
Jaw region	Anterior	50.0%	40	6	85.0%	0.783
	Posterior	50.0%	40	7	82.5%	
Prosthesis type	Single	76.3%	61	11	81.97%	0.438
	Bridge	23.8%	19	2	89.47%	
Surface type	Sandblasted + acid-etched	50.0%	40	4	90.0%	0.235
	Sandblasted	27.5%	22	4	81.8%	
	Laser-modified	22.5%	18	5	72.2%	
Zirconia brand	Zeramex	38.8%	31	2	93.55%	0.252
	Zibone	27.5%	22	4	81.82%	
	Neodent Zi	11.3%	9	2	77.78%	
	Z-Systems	22.5%	18	5	72.22%	
Sex	Female	65.0%	52	7	86.54%	0.345
	Male	35.0%	28	6	78.57%	

Data are expressed as the number of implants placed, failures, and the survival rate (%). Percentages represent the proportion of implants within the analyzed cohort (n = 80). Survival comparisons were performed using the Kaplan–Meier method with log-rank (Mantel–Cox) tests. Bold values indicate statistically significant differences (p < 0.05). Fifteen implants from the initial cohort of 95 were excluded due to loss to follow-up or failure to meet inclusion criteria

**Fig. 1:**
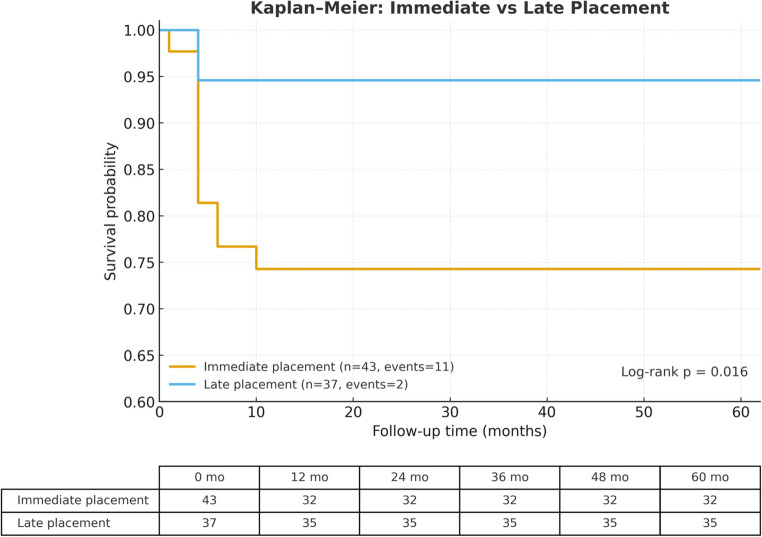
Kaplan–Meier survival curves comparing zirconia implants placed immediately after extraction (*n* = 43, 11 events) and those placed with delayed protocols (*n* = 37, 2 events). The five-year cumulative survival probability was significantly lower for immediate placement (Log-rank test, *p* = 0.016). The table below shows the number of implants at risk at selected follow-up intervals (0, 12, 24, 36, 48, 60 months)

The average follow-up duration was 29.0 ± 16.6 months, with a maximum of 62 months. Kaplan–Meier analysis also showed that the average estimated survival time was 52.6 ± 2.38 months, while the median survival time was not reached due to censoring (Fig. 1). Among these, 58 were two-piece and 22 were one-piece. Over a five-year period, 13 implants experienced failure (16.3%). Of these, four were one-piece implants and nine were two-piece implants. Guided Bone Regeneration (GBR) techniques were employed in 11 surgeries. All implants that underwent Guided Bone Regeneration (GBR) survived, whereas all implant failures occurred in cases without GBR. Kaplan–Meier survival analysis demonstrated a clear separation between the two curves, and the difference in survival between groups was statistically significant (log-rank test, *p* < 0.001).

There was no statistically significant difference between the survival rates of narrow-diameter implants (*n* = 7, no failures) and standard-diameter implants (*n* = 73, 13 failures; *p* = 0.234). A total of 43 implants were placed immediately, and 37 were placed late. Survival analysis showed significantly lower survival for immediate placement compared with late placement (*p* = 0.016). Additionally, no significant differences were found among the 25 implants that were immediately loaded and 47 with delayed loading (*p* = 0.674) (Table 1).

## Discussion

This retrospective study examined the five-year outcomes associated with 80 zirconia implants from an initial cohort of 95, encompassing both one-piece and two-piece systems, as well as cases with and without GBR. The primary objective of the study was to evaluate the survival rate of these implants and to compare outcomes across varying designs, surgical protocols, and grafting procedures. The results revealed a total of 13 implant failures, resulting in an overall survival rate of 83.8%. Notably, the analysis identified a significant difference in survival between immediate and late placement, whereas no significant differences were observed among implant types, loading strategies, brand, surface, or prosthetic design. The present study revealed that all implants placed with GBR remained successful throughout the follow-up period, while every implant failure occurred in sites without GBR, suggesting that adding regenerative techniques may enhance the peri-implant environment and contribute to long-term success stability. These findings indicate that both zirconia implant designs can achieve satisfactory mid-term survival rates in selected patient populations. Consequently, the null hypothesis, which posited the absence of significant differences among the examined variables, was partially rejected, as immediate placement was associated with significantly lower survival.

The current survival rate of 83.8% supports the clinical use of zirconia implants as metal-free options; however, it remains lower than the over 95% long-term survival typically reported for titanium implants [1, 3]. This difference should be interpreted cautiously, as titanium survival data are derived from decades of large-scale prospective studies, whereas zirconia implant systems represent a comparatively newer technology with more limited long-term clinical evidence. Despite this difference in survival benchmarks, zirconia implants offer well-documented biological and esthetic advantages, including superior esthetics [13], favorable soft-tissue integration related to their tooth-colored appearance [14], high biocompatibility [15–18], and reduced bacterial adhesion [19–21]. Their low surface energy further limits bacterial colonization, which may contribute to reduced peri-implant inflammatory responses [22].

 Nevertheless, these biological benefits do not fully offset the technique sensitivity of zirconia implant therapy. The slightly lower survival rate observed in the present cohort may reflect differences in implant design and surface characteristics, as well as a greater sensitivity of zirconia implants to biological and surgical conditions, particularly placement timing, which can critically influence early osseointegration. Reported survival rates for zirconia implants vary widely across studies, ranging from 77.3% [24] to 100% [26], with most five-year investigations reporting outcomes above 90% [23, 25, 27]. Meta-analyses estimate pooled five-year survival rates of approximately 94–95%, with low incidences of implant or abutment fractures and success rates approaching those of titanium implants [27, 28]. The lower survival observed in the present cohort may therefore be partly attributable to the relatively high proportion of immediately placed implants, a protocol that appears to be less predictable for zirconia than for titanium systems. Overall, the results of this study, including the absence of fractures and the observed influence of placement timing, support that zirconia implants can perform reliably in the mid-term when appropriate case selection and surgical protocols are applied. Currently, two zirconia compounds are used for the manufacture of ceramic implants: alumina-toughened zirconia (ATZ) and 3 mol% yttria-doped tetragonal zirconia (Y-TZP). Both ceramic compositions performed similarly in the present cohort, with no significant differences in survival.

The study shows a link between GBR and implant survival, emphasizing the need for adequate bone volume and regenerative support for stable osseointegration with zirconia implants. All implants with GBR survived, but the small sample requires caution. The absence of failures suggests a protective effect, aligning with Benic et al. [29], who found comparable bone formation around zirconia and titanium implants after GBR. Gargallo-Albiol et al. [30] also reported 100% survival of zirconia implants with GBR over 34 months. A recent review by Taghizadeh et al. [31] confirmed GBR’s role in improving implant survival and reducing bone loss, especially in compromised sites. The biological basis may involve better vascularization and space maintenance with grafts, supporting early bone remodeling and load distribution. Overall, these findings highlight the importance of considering GBR in zirconia implant placement, especially in sites with limited bone, to achieve better long-term results.

One-piece ceramic implants have been investigated in several studies and have demonstrated high clinical success and mechanical strength [10, 32]. One-piece implants also offer advantages such as faster surgical procedures, simpler prosthetic approaches, and the absence of a microgap between the abutment and the implant, which prevents the accumulation of microorganisms inside the gap [33, 34]. They have also been reported to be mechanically superior, with lower fracture rates according to in vitro studies [10]. Despite Hashim et al. [14] reporting a higher rate of early implant loss in one-piece ceramic implants, our results showed no statistically significant difference between the survival rates of one-piece and two-piece implant. There are also disadvantages to one-piece implants, such as limitations in adjusting the angulation of the abutment and the necessity for a cemented crown. In contrast, two-piece ceramic implants can accommodate both cemented and screw-retained crowns. Although four of the thirteen failed implants in the present study were one-piece implants, compared with nine two-piece failures, there was no statistically significant difference between groups.

This study presents several limitations that warrant consideration. The lack of comprehensive clinical and radiographic evaluations, including peri-implant bone level measurements and probing depth assessments, limits the ability to assess the health of peri-implant tissues over time thoroughly. Moreover, the retrospective design and relatively modest sample size may influence the generalizability of the findings. Although all failures occurred without GBR in the updated dataset, this finding should be interpreted with caution, as the GBR subgroup was small and may not fully represent broader clinical variability. This outcome is consistent with prior research conducted by Balmer et al. Future prospective studies with larger cohorts and standardized clinical and radiological follow-ups are essential to more accurately ascertain the long-term outcomes of zirconia implants.

## Conclusions

This study reported a 5-year survival rate of 83.8% for zirconia implants, supporting their viability as alternatives to titanium implants. Although clinical parameters such as probing depth and insertion torque were not assessed, implant failures were associated with biological issues rather than mechanical factors, with no fractures observed. These findings indicate good mid-term outcomes for both one-piece and two-piece zirconia implants, although immediate placement showed lower survival rates than late placement. Implants with guided bone regeneration demonstrated consistently higher survival, emphasizing the importance of GBR procedures for long-term success. Future studies involving detailed clinical and radiographic assessments are needed to confirm these results and refine zirconia implant protocols.

## Data Availability

Datasets used and/or analyzed during this study are available from the authors upon reasonable request.
